# Problem gambling among working youth in Southeast Türkiye: predictors and psychological consequences

**DOI:** 10.1186/s12889-026-26878-4

**Published:** 2026-03-04

**Authors:** Mehmet Emin Düken, Özge Karakaya Suzan, Meltem Günaydın

**Affiliations:** 1 Health Sciences of Faculty, Department of Child Health and Diseases Nursing Şanlıurfa, Harran University, Şanlıurfa, 63000 Türkiye; 2 Health Sciences of Faculty, Department of Child Health and Diseases Nursing Sakarya, Sakarya University, Sakarya, Türkiye; 3 Department of Child and Adolescent Psychiatry, Başakşehir Çam and Sakura City Hospital, Istanbul, Türkiye

**Keywords:** Adolescent gambling, Problem gambling, Child labor, Street youth, Depression, Anxiety, Social support, Türkiye

## Abstract

**Background:**

In low-socioeconomic contexts like South-eastern Türkiye, where child labor rates exceed national averages due to economic pressures, working youth face amplified risks of gambling as a maladaptive coping mechanism. The present study was conducted to identify gambling addiction, predictors and psychological consequences among working youth in the South-eastern Anatolia Region of Türkiye.

**Methods:**

This cross-sectional and descriptive study, which was planned in accordance with STROBE guidelines, was conducted between 01.04.2024 and 31.12.2024 with youth working on the streets of Şanlıurfa and Diyarbakır for more than two years. Ethical approval was obtained from the university and institutional permission was obtained from the governor's office in the province where the study was conducted. After explaining the study to each adolescent and parent, informed consent was obtained from all adolescents and parents. The study was conducted with 671 working youth who met the specified inclusion criteria. Regression analysis and mediation models were used in the analyses.

**Results:**

It was found that 18% (122) of the adolescents working on the street played rummikub, 25% (171) played billiards or bowling for money once a week, 32% (217) played iddaa (official football betting game), 17% (112) betted on horse racing, 11% (72) bought numerical lotto tickets, 33% (223) played scratch card games and 23% (152) played online games every week in the last 12 months. Strengths and Difficulties Questionnaire score had a negative statistical effect on multidimensional perceived social support scale (β1 = -0.973), and a positive statistical effect on depressive symptoms (β1 = 0.690) and anxiety symptoms (β1 = 0.726). The change of 60.2% of the scores in the gambling screening test was explained by depressive symptoms, anxiety symptoms and multidimensional perceived social support scores (R^2^ = 0.602). More than half of the gambling screening test was explained by these three variables.

**Conclusions:**

Current evidence indicates that culturally sensitive, enhanced social support could alleviate symptoms of depression and anxiety associated with gambling behaviour. However, randomised controlled trials (RCTs) are needed to establish causality and the clinical efficacy of this relationship definitively. Therefore, future policy frameworks must balance methodological rigour and robust evidence generation with advocacy for the potential role of such interventions.

## Introduction

Gambling is an important public health problem with adverse effects on individuals, their families, close associates and the wider society [1, 2]. Despite implementation of legislations worldwide to protect youth against gambling, online betting and emergence of smartphones and gambling related applications led to increased risks for youth gambling [1, 3]. Earlier studies found that 0.2–12.3% of youth may meet the criteria for problem gambling while a recent meta-analysis of 366 studies found that 17.9% (95% Confidence Interval: 14.8–21.2) of adolescents (mostly males) worldwide had gambled within the past month [2, 3]. Rates of adolescent problem gambling are thought to equal those among adults. Adolescent problem gambling may also be associated with delinquency, criminal behaviour, poor academic achievement, truancy, financial problems, depressive symptoms, suicidality, lower self-esteem, social problems and substance abuse as well as culminating in adult gambling underlining its importance for preventive interventions [1]. However, while global estimates are substantial, national prevalence varies significantly, and data on vulnerable subgroups remain sparse. For instance, in Turkey, reported problem gambling rates among youth are lower (e.g., 2.9%) [4], yet specific high-risk populations such as street-working adolescents—who face compounded socioeconomic stressors—are critically understudied. This gap is significant, as prior research on labor conditions suggests that such stressors may elevate behavioural health risks by 2–threefold.

Within this broader framework, regional socioeconomic disparities in Türkiye provide critical context for understanding child labor and gambling vulnerability among working adolescents. Socioeconomic disparities across regions of Türkiye constitute a critical contextual factor influencing child labor and related psychosocial vulnerabilities. National statistics consistently show that household income levels in southeastern regions of Türkiye are substantially lower than those in western regions, reflecting long-standing structural inequalities [5]. Persistent poverty has been associated with higher fertility rates, as families may perceive larger household size as a strategy to enhance socioeconomic security; however, increasing numbers of children under conditions of economic deprivation often exacerbate financial strain rather than alleviate it [6]. This dynamic significantly elevates the risk of child labor, particularly street-based economic activities [7]. In this context, begging should not be conceptualized as a form of service-sector employment, but rather as a behavioral coping strategy through which individuals seek financial support to meet basic needs, frequently appealing to emotional sensitivities and prevailing social norms of compassion [8].

Problem gambling among adolescents is accepted to evolve within the context of a dynamic interplay of risk and protective factors [1, 9]. Risk factors may include male gender (OR = 25 or 37), positive attitudes of parents/peers towards gambling (OR = 3.0), alcohol/substance use, novelty seeking/impulsivity (OR = 2.0), reduced social connectedness, increased access to gambling activities, financial incentives and viewing gambling as an activity to regulate emotions. Elevated social connectedness, greater prosocial behaviour, positive relationships with parents, participation in meaningful extracurricular activities are listed among protective factors while immigrant youth, out-of-school adolescents, working youth, and transgender/gender diverse adolescents may form vulnerable groups [1, 3]. Developmental and cognitive immaturity, increased risk taking and susceptibility to peer influences and marketing campaigns may be especially important for adolescent gambling [9]. Crucially, research indicates that temperamental traits (e.g., impulsivity) can significantly amplify the risks posed by environmental exposures (e.g., access, peer norms), particularly in high-stress contexts such as those experienced by working youth. This multifactorial and interactive model underscores the necessity of research that examines not only the prevalence of risk factors but also their synergistic effects within specific vulnerable populations.

Data on problem gambling among Turkish youth are limited [4, 10–12]. Aricak's school-based survey (*n* = 6116) reported 12.4% online gambling, likely underestimating risks in unschooled laborers [4]. Koksoy Vayisoglu et al. (2019) found in a college sample that 41.4% have gambled at least once in lifetime, 21.5% in the past month and 15.3% gambled online. Pathological gambling in this study was found in 1.2% of the students [11]. In an earlier study Bayar and Sayil (2005) found that 13.0% of high school and college students living in Ankara had gambled, at least occasionally [10]. Adolescent employment was found to be associated with problem gambling, substance use and elevated rates of psychopathology in several studies from Turkey as well as other countries [13–19]. However, the effects of work may depend on length of working hours, level of social/financial supports as well as exposure to abuse at work and employment status may interact with temperamental factors of the adolescents to increase the risk of psychopathology, including problem gambling [14, 18, 19]. Previous studies on psychopathology among working Turkish youth focused on adolescents attending vocational training centres, those living in western regions of Turkey and focused on symptoms of general psychopathology [16, 17]. Therefore, this study aimed to evaluate;

- A. Estimate past-year gambling rates and frequencies;
- B. Examine motives as mediators between psychosocial stressors and participation;
- C. Assess associations with depressive/anxiety symptoms;
- D. Identify demographic predictors via regression.
- E. To integrate the above objectives within a comprehensive analytical framework by modeling the direct and indirect pathways between psychosocial problems, perceived social support, and gambling severity using multivariable regression and path analysis.

## Material-method

### Study design, setting

This study was conducted as a cross-sectional study between 01.04.2024 and 31.12.2024 on youth working on the streets of Şanlıurfa and Diyarbakır for more than two years. Median income is lower in the south-eastern region of Türkiye compared to the western regions. This is thought to cause families to conceive and give birth at frequent intervals in order to increase their socioeconomic level. Increasing number of children and low socioeconomic status in the region increase the prevalence of child labour [20]. The populations of Şanlıurfa and Diyarbakır are estimated to be 2.2 and 1.8 million, respectively, while the proportion of children under 18 in these cities is reported to range between 18.0- and 20.0% and 16.0 and 18.0%, respectively [5]. The study was conducted with adolescents working in various workplaces (bakery, supermarket, greengrocer, tailor, barber, café, haberdashery, auto industry, streets and parks) in these cities.

### Study participants and sampling

The adolescents in the study were working on the streets, in industry and service sectors in Şanlıurfa and Diyarbakır metropolitan cities. The adolescent workers in the study sample were working in service sectors such as bakeries, supermarkets, grocery stores, tailors, barbers, cafes, haberdashery, as beggars on the streets and parks, and in the auto industry. Data were collected from the adolescents working in the central districts of the provinces between the determined dates by using the snowball sampling method (Non-Probability). To mitigate inherent biases such as network homogeneity and referral bias, recruitment employed diversity monitoring across chains and ceased at theoretical saturation (*n* = 671). These measures, aligned with tailored design principles for mixed-methods research, enhance methodological reflexivity when studying interconnected populations [21–23].

In the post-power analysis conducted to determine the medium-level effects (f^2^ = 0.15) in multiple regression with nine predictors, the test power was obtained as 90% (1-β) with a sample size of 85% power. The sample size was found to be adequate in the study conducted with 671 adolescents.

Inclusion criteria included being between 14- and 19-years of age, working as an adolescent worker in the informal/service sector on the street for ≥ 2 years, being able to read and write at a third-grade level, working as an adolescent worker in designated cities, and volunteering to participate in the study. Adolescents under 14 years of age, adults over 19 years of age, illiterate adolescents, adolescents with chronic medical conditions including speech/vision/hearing problems, adolescents who refused to participate, and adolescents whose parents/legal guardians did not give informed consent were excluded.

### Data collection

Adolescent Follow-up Form was used to obtain demographic data from working youth, South Oaks Gambling Screening Test-Revised Adolescent Form was used to determine gambling problem levels, Strengths and Difficulties Questionnaire and Internalised Behaviour in Youth Scale were used to determine psychological problems, and Multidimensional Perceived Social Support Scale was used to determine social support perceived by adolescents from family, friends and significant others.

### Administration

The data were collected from adolescents working in various jobs in the districts of two cities in south-eastern Turkey. Each adolescent worker and their parents were informed about the study and included in the study after verbal consent and written informed consent were obtained. Adolescent workers and their parents were interviewed during working hours and the study data were completed by the adolescents. For third-grade literacy participants, scales were read verbatim without interpretive input, per standardized administration to preserve response authenticity.

### Data collection tools

#### Adolescent follow-up form

The relevant information on the sociodemographic characteristics and problems experienced by adolescents working in various jobs in the central districts of the cities in the Southeast of Turkey was compiled by the researcher by reviewing the literature [24]. This form included questions about the age, education level, mother’s education level, father’s education level, years of schooling, number of siblings, and monthly income of adolescents. In order to determine the characteristics related to their work and the reasons for gambling, questions such as the number of gambling sessions per week, the number of gambling sessions per month, the number of gambling sessions per year, the gambling status of friends around them, and the gambling status of their parents were asked.

#### South Oaks Gambling Screen – Revised for Adolescents (SOGS-RA)

The SOGS-RA is one of the most frequently used inventories to provide estimates of the societal prevalence and lifetime prevalence of problem gambling among adolescents [25]. A form adapted to adolescents was developed and a Turkish validity and reliability study was conducted. The scale measures whether adolescents have lifetime participation in various types of gambling (card games, rummikub, coin tossing, bowling/billiards, betting on the results of sports competitions, horse races, dice, number ten/numeric lotto/super lotto/lucky ball, instant win, bingo, national lottery, internet gambling and others) and how often they have gambled in the last 12 months [26, 27]. The cut-off score of the scale is 8. In the present sample, the Cronbach’s alpha value of the SOGS-RA was 0.89.

#### Youth Internalizing Behaviour Screener – YIBS

The YIBS was developed to identify symptoms of anxiety and depression in children and adolescents. The scale consists of 10 items and each item is four Likert-type and scored between 10 and 40 points. The higher the score on the scale, the higher the symptoms of depression and anxiety (internalized behaviours) [28]. In the present sample, the Cronbach’s alpha value of the YIBS was 0.85.

#### Strengths and Difficulties Questionnaire (SDQ)

The SDQ is used to determine the emotional, conduct, peer, attention deficit hyperactivity and prosocial behaviours of children and adolescents. The scale consists of 25 items and each item is answered between 0 and 2 and scored between 0 and 40 [29, 30]. In the present sample, the Cronbach’s alpha value of the SDQ was 0.81. Emotional problems score was evaluated as normal between 0 and 3, borderline when 4 and abnormal between 5 and 10. Behavioural problems score was considered normal between 0 and 2, borderline when 3, and abnormal between 4 and 10. Peer problem score was evaluated as normal between 0- and 2, borderline when 3, and abnormal between 4 and 10. Attention deficit and hyperactivity score was evaluated as normal between 0 and 5 points, borderline when 6 points and abnormal between 7 and 10 points. Total mean scores of 0–13 points were considered normal, 14–16 points were considered borderline and 17–40 points were considered abnormal. The mean score for prosocial behaviours was considered normal between 6- and 10-points, borderline when 5, and abnormal between 0 and 4 points. Permissions to use the scale have been obtained.

#### Multidimensional Perceived Social Support Scale (MSPSS)

The MSPSS was used to determine the social support perceived by working adolescents from peers, significant others and their families. The scale consists of 12 items, each item is answered on a seven-point Likert scale and ranges between 12 and 84 points. Higher scores indicate better perceived social support [31, 32]. In the present sample, the Cronbach’s alpha value of the MSPSS was 0.88.

### Data analysis

In the study, Skewness and Kurtosis values were examined with SPSS 22 (Statistical Program in Social Sciences) program to evaluate whether the data were normally distributed. Reliability analysis and multicollinearity analysis were performed with the SPSS program. Quantitative data were expressed as means and standard deviations (SD) or medians (min–max) depending on normality and outlier assumptions. Categorical data were reported as number and frequency. *P* value for significance was set at 0.050.

Structural equation modelling analysis was applied by establishing a multivariate linear regression model to explain the correlations between the data with a mathematical equation by providing the assumptions of multivariate analysis, making the first observation of the correlations between the scales and building models for the correlations between the scales by using the AMOS 23 program according to the correlations. PATH analyses were performed according to the types of correlations.

First, an observed-variable path analysis model was specified and tested in AMOS. All study variables were included in the model as manifest variables, and no latent constructs were defined. Model fit was evaluated using multiple fit indices. Although a CMIN/df value below 3 is considered indicative of good fit, values below 5 are widely accepted as representing acceptable model fit in applied psychosocial research. Accordingly, the fit indices indicated an acceptable fit of the proposed path model (CMIN = 265.790, df = 54, CMIN/df = 4.92, RMSEA = 0.079, CFI = 0.969, NFI = 0.967, GFI = 0.925, IFI = 0.969, RFI = 0.943, TLI = 0.947).

## Results

It was found that 58.6% of the adolescents working on the streets were male, 16.4% had a deceased mother and 15.6% had a deceased father. It was found that 31.3% of the adolescents’ mothers were illiterate, 31% of the adolescents’ fathers were illiterate and 59.9% of the adolescents had high school education. It was found that 77.9% of adolescents smoked cigarettes, 43.8% smoked narghiles and 35.8% used energy drinks daily (Table 1).

**Table 1:** Distribution and mean scores of demographic characteristics of adolescents working on the streets

		n	%
Gender	Female	278	41.4
	Male	393	58.6
Is the mother alive	Yes	561	83.6
	No	110	16.4
Is the father alive	Yes	566	84.4
	No	105	15.6
Mother’s educational status	Illiterate	210	31.3
	Elementary	101	15.1
	Secondary	107	15.9
	High school	143	21.3
	Deceased	110	16.4
Father’s educational status	Illiterate	208	31.0
	Elementary	78	11.6
	Secondary	132	19.7
	High school	148	22.1
	Deceased	105	15.6
Adolescent’s educational Status	Illiterate	120	17.9
	Secondary	149	22.2
	High school	402	59.9
Smoking status	Yes	523	77.9
	No	148	22.1
Narghile smoking status	Yes	294	43.8
	No	377	56.2
Status of using energy drinks	Yes	240	35.8
	No	431	64.2

	Mean ± SD	Median (Min–Max)	
Adolescent’s age	16.16 ± 1.38	16 (14—19)	
Years of working	6.00 ± 2.47	5 (2—13)	
Number of siblings	6.59 ± 2.84	5 (1—14)	
Daily working hours	7.63 ± 2.08	7 (4—12)	
Daily sleeping hours	7.74 ± 1.1	8 (6—10)	
Duration of Daily Smartphone Use	4.33 ± 0.81	4 (3—7)	
Number of Cigarettes Smoked Daily	18.11 ± 9.33	20 (4—35)	
Number of Narghiles Smoked per Month	15.97 ± 4.81	17(1—23)	
Number of Energy Drinks Drank Per Day	1.85 ± 1.24	1 (1—5)	

The average age of the adolescents who participated in the study was 16.16 ± 1.38 years, they had been working for an average of 6.00 ± 2.47 years, and they had an average of 6.59 ± 2.84 siblings. It was found that adolescents worked 7.63 ± 2.08 h daily, slept 7.74 ± 1.10 h daily and used smartphones for 4.33 ± 0.81 h daily. It was found that adolescents smoked 18.11 ± 9.33 cigarettes daily, they smoked narghile an average of 15.97 ± 4.81 times a month and drank 1.85 ± 1.24 energy drinks daily (Table 1).

The sociodemographic profile of the sample indicates marked socioeconomic disadvantage, characterized by high parental illiteracy, early and prolonged labor participation, and limited educational attainment. These baseline vulnerabilities provide important context for interpreting the high prevalence and severity of gambling behaviors observed in subsequent analyses.

100% (671) of the adolescents stated that they had never played card games for money, tossed a coin for money, played dice games for money, bingo for money and none of the unspecified types of gambling during their lifetime. It was reported that 69% (463) of the adolescents played rummikub for money at least once in their lives, 51.9% (348) bet on games that require personal skills such as billiards and bowling, 77.9% (523) played iddaa, 53% (355) betted on horse racing, 44. 4% (298) reported that they bought numerical lotto, lottery, super lotto tickets, 77.5% (520) played scratch-off, 59% (396) bought national lottery tickets and 77.9% (523) gambled on the internet (Table 2).

**Table 2:** Types and distribution of lifetime and last twelve months gambling among adolescents

	Lifetime	Last Twelve Months			
	Never	At least once	Less than once a month	Once a month	Once a week
Playing Card Games for Money	671 (100%)	0(0%)	0(0%)	0(0%)	0(0%)
Playing rummikub for Money	208(31%)	463 (69%)	50(7%)	291(43%)	122(1%8)
Tossing a coin for money	671 (100%)	0(0%)	0(0%)	0(0%)	0(0%)
Betting on Games that Require Personal Skill such as Billiards, Bowling	323 (48.1%)	348 (51.9%)	19(3%)	158(24%)	171(25%)
Playing Iddaa	148(22.1%)	523(77.9%)	62(9%)	244(36%)	217(32%)
Betting on Horse Racing	316(47%)	355(53%)	77(11%)	166(25%)	112(17%)
Playing Dice Games for Money	671 (100%)	0(0%)	0(0%)	0(0%)	0(0%)
Buying Numerical Lotto, Luck Ball, Super Lotto Tickets	373(55.6%)	298(44.4%)	34(5%)	192(29%)	72(11%)
Playing Scratchers	151(22.5%)	520(77.5%)	56(8%)	241(36%)	223(33%)
Playing Bingo for Money	671 (100%)	0(0%)	0(0%)	0(0%)	0(0%)
Buying a National Lottery Ticket	275(41%)	396(59%)	396(59%)	0(0%)	0(0%)
Gambling on the Internet	148(22.1%)	523(77.9%)	47(7%)	324(48%)	152(2%3)
Unspecified Forms of Gambling	671 (100%)	0(0%)	0(0%)	0(0%)	0(%0)

^*^Lifetime and past 12-month participation rates for specific gambling activities among working adolescents. Percentages represent activity-specific engagement and are not mutually exclusive; therefore, individuals may have participated in multiple forms of gambling. The table should be interpreted as reflecting patterns of polygambling rather than unique or cumulative prevalence

It was found that 18% (122) of the adolescents working on the street played rummikub for money once a week in the last 12 months. It was found that 25% (171) of the adolescents bet on games that require personal skills such as billiards and bowling in the last 12 months. It was determined that 32% (217) of the adolescents played iddaa and 17% (112) betted on horse racing once a week in the last 12 months. It was reported that 11% (72) of the adolescents bought numerical lotto, luck ball, super lotto tickets and 33% (223) played scratch card games every week in the last twelve months. It was found that 23% (152) of the adolescents played online games every week in the last twelve months (Table 2). Because gambling activities were assessed individually, overlapping participation across multiple gambling types was common, indicating a pattern of polygambling rather than isolated engagement in single activities.

77.9% (523) of the adolescents who worked on the street stated that one of their parents played games of chance and 76% (510) of them stated that it was their father. 70.9% (476) of the adolescents stated that their parents gambled a lot and 69.2% (464) of the adolescents stated that their fathers gambled a lot. 10.1% (68) of the adolescents always gambled and 27.3% (183) of the adolescents gambled most of the time. Adolescents reported that 24.6% (165) borrowed money from relatives, 77.9% (523) borrowed money from friends, and 27.6% reported that they stole money to gamble. According to the mean score of the South Oaks Gambling Screening Test Score of adolescents, individuals with a score higher than 8 points were defined as pathological gamblers. It was determined that 76.8% (515) of the adolescents were pathological gamblers. It was determined that the maximum amount that adolescents played for gambling per day was 1102.09 ± 780.65 TL (Table 3).

**Table 3:** Gambling related variables of adolescents working on the street

Gambling Related Variables		n	%
Does any of the parents play chance games	No	148	22.1
	Yes	523	77.9
Which parent?	Father	510	76.0
	Both mother and father	13	1.9
	None	148	22.1
Do you think of which parent gambles too much	No	195	29.1
	Yes	476	70.9
The parent who gambles too much	Father	464	69.2
	Both mother and father	12	1.6
	None	195	29.2
Gambling Frequency	Always	68	10.1
	Most of the time	183	27.3
	Sometimes	272	40.5
	Neve	148	22.1
Do you borrow money from relatives for gambling	No	506	75.4
	Yes	165	24.6
Do you borrow money from friends for gambling	No	148	22.1
	Yes	523	77.9
Do you steal for gambling	No	486	72.4
	Yes	185	27.6
Number of Pathological Gamblers in terms of Gambling Scores	Non-pathological Gamblers	156	23.2
	Pathological Gamblers	515	76.8
	Mean ± SD	Median (Min–Max)	
Maximum Daily Amount of Gambling (TL)	1102.09 ± 780.65	1100 (0—3000)	

It was found that the mean depression score of adolescents working on the street was 11.34 ± 3.29, their mean anxiety score was 10.95 ± 2.69, and their mean internalized youth behaviour score was 22.29 ± 5.71. The mean emotional problem score of adolescents was 6.05 ± 2.69, the mean behavioural problems score was 6.04 ± 2.29, the mean hyperactivity score was 6.08 ± 2.7, the mean peer problems score was 6.02 ± 2.35 and the mean prosocial score was 6.18 ± 2.47. The mean total strength and difficulties score was 24.19 ± 9.29, the mean externalized score was 12.17 ± 4.81 and the mean internalized score was 12.07 ± 4.77. The mean score of Gambling Addiction of adolescents was found to be 8.98 ± 4.98. The mean score of social support perceived by adolescents from their environment was 45.99 ± 24.79 (Table 4).

**Table 4:** Mean scale scores of adolescents working on the streets

**Variables**	Cut-off Values	Scale Min–Max Values	Mean ± SD	Median (Min–Max)
Depression	12	4–20	11.34 ± 3.29	11 (6—18)
Anxiety	11	4–20	10.95 ± 2.69	11 (7—19)
Youth Internalizing Behavior Screener	23	8–40	22.29 ± 5.71	22 (14—35)
Emotional Problems Scale	5–10	0–10	6.05 ± 2.69	7 (0—10)
Conduct Problems Scale	4–10	0–10	6.04 ± 2.29	7 (0—10)
Hyperactivity Scale	7–10	0–10	6.08 ± 2.7	7 (0—10)
Peer Problems Scale	4–10	0–10	6.02 ± 2.35	6 (1—10)
Prosocial Scale	0–4	0–10	6.18 ± 2.47	7 (1—10)
Total Difficulties Score	17–40	0–40	24.19 ± 9.29	28 (5—37)
The Externalizing Score (Conduct Problems and Hyperactivity Scale)	-	0–20	12.17 ± 4.81	14 (2—28)
The Internalizing Score (Emotional and Peer Problems Scale)	-	0–20	12.07 ± 4.77	14 (2—19)
South Oaks Gambling Screening Test Score	8	0–12	8.98 ± 4.98	8 (0—12)
Multidimensional Perceived Social Support Scale	-	12–84	45.99 ± 24.79	41 (12—84)

Strengths and difficulties questionnaire has a negative effect on multidimensional perceived social support scale (β1 = −0.973; *p* < 0.001) and a positive statistical effect on depression symptoms (β1 = 0.690; *p* < 0.001) and anxiety symptoms (β1 = 0.726; *p* < 0.001). Strengths and Difficulties Questionnaire has the highest effect on multidimensional perceived social support scores and the lowest effect on depression symptoms scores (Table 5).

**Table 5:** Structural equation modelling results

Explained variable		Explaining variable	B_1_	B_2_	Standard Error	Test Sta	*P*	R2
Depression	< –-	SDQ	0.690	1.06	0.049	21.431	*P* < 0.001	0.476
Anxiety	< –-	SDQ	0.726	0.911	0.039	23.138	*P* < 0.001	0.527
MSPSS	< –-	SDQ	−0.973	−11.27	0.273	−41.305	*P* < 0.001	0.947
Emotional Problems	< –-	SDQ	0.897	1.125	0.033	33.949	*P* < 0.001	0.805
Conduct Problems	< –-	SDQ	0.911	0.977	0.028	35.3	*P* < 0.001	0.831
Hyperactivity Scale	< –-	SDQ	0.907	1.144	0.033	34.868	*P* < 0.001	0.822
Peer Problems	< –-	SDQ	0.921	1.011	0.028	36.169	*P* < 0.001	0.849
Prosocial Scale	< –-	SDQ	0.868	1				0.753
SOGS-RA	< –-	Depression	0.058	0.087	0.041	2.136	0,033	0.609
SOGS-RA	< –-	Anxiety	0.044	0.082	0.053	1.539	0.044	
SOGS-RA	< –-	MSPSS	−0.993	−0.199	0.006	−31.578	*P* < 0.001	

*SDQ* Strengths and Difficulties Questionnaire, *SOGS-RA*:South Oaks Gambling Screning, *MSPSS* Multidimensional Perceived Social Support Scale

*p < 0,05; t test result for the significance of the regression coefficients

Β_1_ Standardized regression coefficients

β_2_ Unstandardized regression coefficients

Strength and difficulties questionnaire positively affects emotional problems (β1 = 0.897; *p* < 0.001), conduct problems (β1 = 0.911; *p* < 0.001), attention deficit and hyperactivity scores (β1 = 0.907; *p* < 0.001), peer problems (β1 = 0.921; *p* < 0.001) and prosocial problems (β1 = 0.868; *p* < 0.001) in a statistically significant way. Strengths and Difficulties Questionnaire affects peer problems at the highest level and prosocial problems at the lowest level (Table 5).

Depression symptoms have a statistically significant positive effect on gambling screening test scores (β1 = 0.058; *p* = 0.001). Anxiety symptoms have a statistically significant positive effect on gambling screening test scores (β1 = 0.044; *p* = 0.001). Multidimensional perceived social support score has a negative and statistically significant effect on gambling screening test scores (β1 = −0.993; *p* = 0.001) (Table 5).

**Fig. 1:**
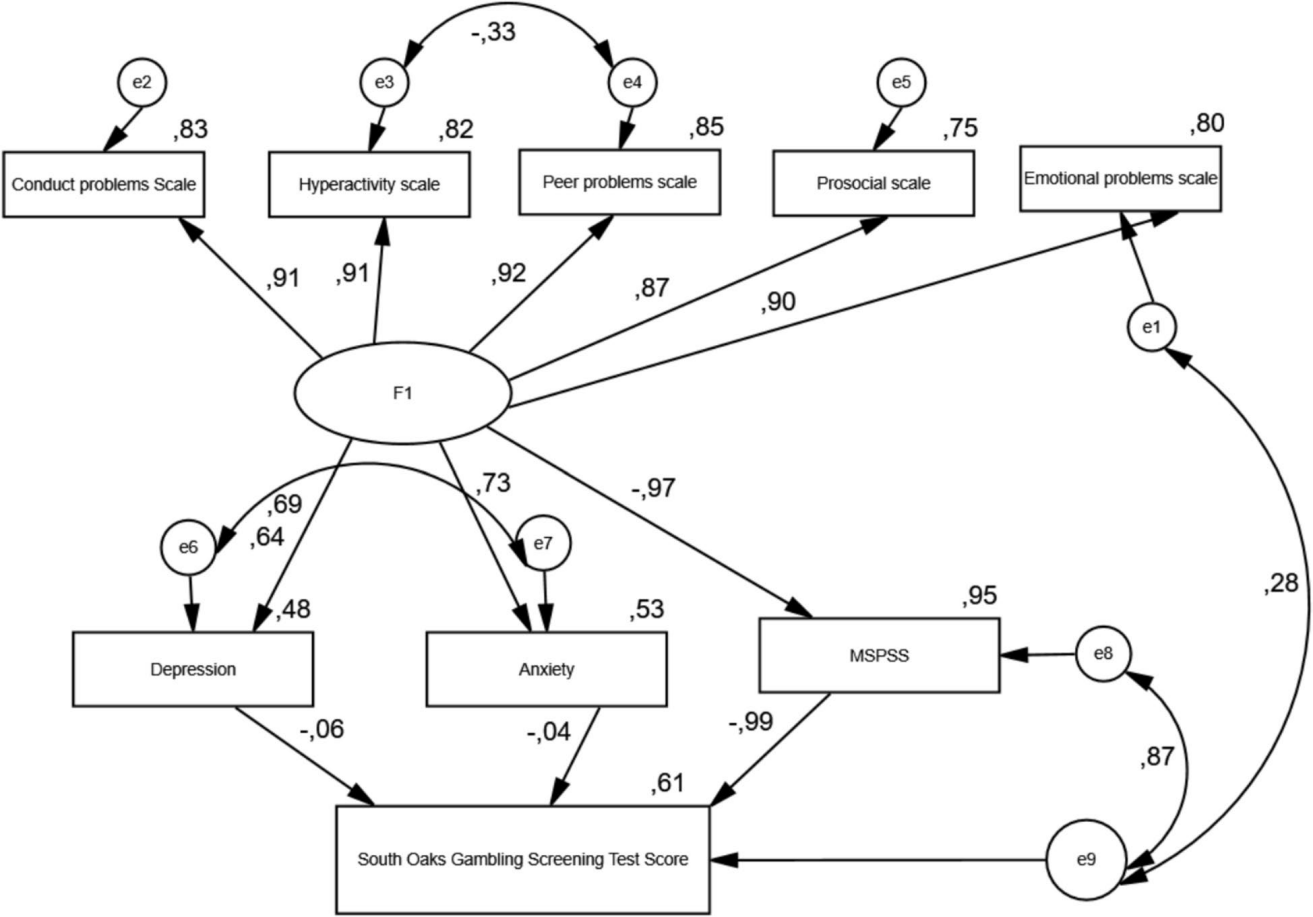
Standard coefficients

60.2% of the change in scores of the gambling screening test was explained by scores on depression symptoms, anxiety symptoms and multidimensional perceived social support (R^2^ = 0.602). 47.6% of the change in depression symptom score is explained by the strengths and difficulties questionnaire score (R^2^ = 0.476). 52.7% of the change in the anxiety symptom score is explained by the score of the strengths and difficulties questionnaire (R^2^ = 0.527). 94.7% of the change in the multidimensional perceived social support score is explained by the score of the strengths and difficulties questionnaire (R^2^ = 0.947) (Table 5) (Fig. 1).

The hierarchical stepwise multiple regression model for the gambling screening test of working adolescents was found to be statistically significant (F = 579.080; *P* < 0.001). Variables such as age, number of siblings, daily sleeping hours, number of cigarettes smoked, number of narghiles smoked, internalized score (behaviour and hyperactivity problems) and externalized score (Emotional and Peer problems) were found to explain 87.3% of the variation in Gambling screening test scores of working adolescents (R^2^ = 0.873) (Table 6). Demographic and behavioral variables were entered first, followed by psychosocial variables, in line with the theoretical framework. The final model explained a substantial proportion of variance in gambling scores (Adjusted R^2^ = 0.873), with no evidence of problematic multicollinearity (all VIF values < 5).

**Table 6:** Multiple regression model for the effect of some demographic characteristics of working adolescents on gambling screening test score (stepwise)

Değişken	B(%95)	Beta	t	p	Zero-order	Partial	VIF
(Constant)	0,647 (−2,463—3,758)		0,409	0.683			
The Internalising Score (Emotional and Peer Problems Scale)	0,631 (0,559—0,702)	0,604	17,403	P < 0.001	0,895	0,560	6,375
Number of Narghiles Smoked	−0,169 (−0,201—−0,138)	−0,291	−10,552	P < 0.001	0,449	−0,379	4,013
Number of Cigarettes Smoked	0,173 (0,142—0,205)	0,388	10,818	P < 0.001	0,665	0,388	6,818
Adolescent’s age (Years)	−0,334 (−0,463—−0,205)	−0,093	−5,083	P < 0.001	0,038	−0,194	1,769
The Externalising Score (Conduct Problems and Hyperactivity Scale)	0,193 (0,12—0,267)	0,187	5,190	P < 0.001	0,851	0,198	6,865
Amount of gambling (TL)	0,001 (0,001—0,001)	0,163	6,346	P < 0.001	0,725	0,239	3,493
Daily sleeping time (Hours)	0,307 (0,088—0,527)	0,068	2,756	0.006	−0,373	0,106	3,206
Number of siblings (person)	−0,155 (−0,273—−0,037)	−0,088	−2,569	0.010	0,472	−0,099	6,252

B(%95): Unstandardised Coefficients, Beta: Standardised Coefficients, Adj R2:0.873, F:579.080, *P* < 0.001, S.E: 1.771, Predictors were entered using a theory-driven hierarchical stepwise regression approach. Demographic and behavioral variables were entered in the first step, followed by psychosocial variables in subsequent steps. Multicollinearity diagnostics indicated no significant violations (maximum VIF < 5). The final model demonstrated strong explanatory power (Adjusted R^2^ = 0.873)

## Discussion

Although gambling is traditionally considered primarily an adult problem, expansion of gambling industry has led to a significant increase in gambling among adolescents. Substantial evidence highlights the emergence of youth gambling behaviour as a public health problem [33].

The panorama of gambling has changed significantly in recent decades, evolving from an initially mild form of entertainment to a dangerous addiction that leads to a range of academic, behavioural, social, financial, criminal or mental health problems for children and adolescents who experience problems with gambling [34–36]. The present study examined the prevalence of gambling among adolescents and the factors affecting this prevalence. The findings suggest that various types of gambling are prevalent among adolescents and this may lead to important psychosocial consequences. Derevensky and Gilbeau (2015) stated that typical forms of gambling among young people included: playing cards (poker) for money, sports betting, dice and board games with family and friends; betting on games requiring personal skill (e.g. billiards, bowling, basketball) with peers; arcade or video games for money; buying lottery tickets; betting on horse and dog races; gambling in bingo halls and card rooms; playing slot machines and table games in casinos; gambling at video lottery/poker terminals; betting over the Internet; and more recently betting at a bookmaker, mostly via the Internet or smartphones [37]. Our results showed that although 100% of the adolescents stated that they did not play traditional forms of gambling (e.g., card games for money, coin toss, dice, etc.) during their lifetime, 77.9% (523) of them played İddaa, 53% (355) betted on horse racing, 44. 4% (298) bought numerical lotto, lucky ball, super lotto tickets, 77.5% (520) played scratch-off, 59% (396) bought national lottery tickets and 77.9% (523) gambled on the internet. Andrie et al. (2019), in their study with 13,284 students aged 14–18, reported that 6% of the study sample reported gambling online (in any form) in the last year, 10% reported gambling offline, and 12.5% reported gambling in any setting [38]. In their study conducted with 6,116 students between the ages of 12 and 18, Arıcak (2019) reported that 12.4% of adolescents bet online [4]. It has been found in the literature that adolescents gambled within a range of 0.6%−38.1% [39, 40]. The finding of gambling online or over the internet suggests that gambling behaviours have changed over time and new types of gambling have become more common among adolescents, especially with the effect of digitalisation. This shift is particularly salient in regions with high digital accessibility; for instance, in contexts where youth smartphone penetration exceeds 90% [41], studies indicate a two-fold higher odds of engaging in digital versus offline gambling, thereby grounding the observed 77.9% internet gambling rate in measurable infrastructure. Especially the high rate of 77.9% gambling over the internet shows the effect of technology and digital gaming environments on gambling behaviour, with anonymity and accessibility serving as key amplifying mechanisms. This finding is also supported in the literature. Studies show that internet gambling becomes attractive for adolescents due to its accessibility and anonymity and increases the risk of addiction [42]. It was also found that 77.5% of adolescents were inclined towards traditional games of chance such as scratch-offs and 59% of adolescents were inclined towards traditional games of chance such as buying national lottery tickets. This suggests that even legal games of chance have the potential to create addiction and are a gateway especially for adolescents.

The present study showed that adolescents working on the streets had high rates of gambling on a weekly basis and their parents also had high rates of gambling. In particular, 77.9% of the adolescents working on the street reported that at least one of their parents gambled and a large number of them reported that their fathers gambled, suggesting that parental gambling habits may increase the risk of gambling in adolescents. In particular, While parental habits predict adolescent gambling (*r* = 0.20), bidirectional effects and SES confounds warrant family-level interventions [43]. Previous research also suggests that parental modelling shapes gambling behaviour in adolescents and that parents’ gambling habits are an important factor that increases the likelihood of their children gambling [4, 43–45]. These findings are consistent with prior evidence indicating that family-related factors, including parental addictive behaviors and dysfunctional family environments, constitute central determinants of adolescents’ tendency toward addictive behaviors. In a comprehensive review of adolescent girls, family functioning, parental substance use, and adverse home environments were identified as key predisposing factors for addiction vulnerability [46]

Almost half of online gambling activities now take place through mobile applications, made possible by the prevalence of smartphones among young people. Online products are also more addictive than traditional forms of gambling [47, 48]. This has increased the number of young pathological gamblers and the growth of online gaming. The number of pathological gamblers in our study was 76.8%. The prevalence of pathological gambling identified in the present study (76.8%) is substantially higher than rates reported in population-based studies of adolescents worldwide and in Türkiye, and therefore requires cautious interpretation. Global prevalence estimates for adolescent problem gambling typically range between 0.2% and 12.3%, while recent meta-analytic evidence suggests that approximately 17–18% of adolescents engage in gambling behaviors within a given month, with markedly lower proportions meeting criteria for pathological gambling [2, 3]. Studies conducted in Türkiye among school-based or university samples similarly report considerably lower prevalence rates of pathological gambling [4, 11]. This discrepancy can be largely attributed to the distinctive characteristics of the study population and the sampling strategy employed. Unlike community- or school-based surveys, the present study exclusively focused on adolescents engaged in informal and street-based labor in socioeconomically disadvantaged urban contexts, recruited using a non-probability snowball sampling method. Snowball sampling is particularly effective for accessing hidden or vulnerable populations; however, it may result in the overrepresentation of individuals with shared environmental exposures and clustered high-risk behaviors, thereby inflating prevalence estimates compared to probability-based samples [22]. It is also important to consider the measurement properties of the South Oaks Gambling Screen–Revised for Adolescents (SOGS-RA). While the SOGS-RA is widely used in epidemiological research, it is sensitive to frequent gambling engagement and cumulative exposure to gambling-related behaviors, particularly in contexts where gambling is socially normalized within family and peer environments [3, 27]. In high-risk samples such as working adolescents, this sensitivity may lead to higher classification rates of pathological gambling compared to general adolescent populations.

In-game gambling, such as playing casino games and opening loot boxes, is becoming increasingly common in apps, online video games and unlicensed third-party websites, with some children asking their parents for pocket money specifically to be spent on these gambling games [49]. In the present study, the maximum amount that adolescents gambled daily was 1102.09 ± 780.65 TL. It is also noteworthy that adolescents tend towards risky behaviours such as borrowing and stealing to gamble. It was found that 77.9% of the adolescents provided financial resources by borrowing from friends, 24.6% by borrowing from relatives and 27.6% by stealing to gamble. This suggests that gambling addiction can trigger criminal behaviours in adolescents and lead to more risky behaviours by increasing financial distress [50]. It shows that gambling addiction is not only limited to economic losses, but also negatively affects the psychosocial functioning of individuals [51].

Gambling behaviour was also found to be related to psychological factors in the present study. In particular, 76.8% of adolescents were classified as pathological gamblers according to the South Oaks Gambling Screening Test (SOGS), indicating that gambling addiction is a serious problem in this age group. It was determined that adolescents who gambled had high scores of depression, anxiety and internalised conduct disorder. Although the current scientific literature on problematic online gambling is still limited [52], it has been emphasised that it can lead to significant consequences. For example, it has been noted to lead to numerous mental health problems, including depression, stress and anxiety [42, 53–55]. In particular, longitudinal research has revealed several modifiable psychosocial factors that are consistently positively associated with the development of problem gambling, including depression, anxiety, hazardous alcohol use, weekly tobacco use and poor general health [56, 57]. Gambling disorders have also been associated with various mental health problems such as high levels of impulsivity, anxiety, depression and stress [58–60]. In the present study, 60.2% of the change in gambling screening test scores was explained by depression symptoms, anxiety symptoms and multidimensional perceived social support scores (R^2^ = 0.602). While it has been emphasised in the literature that social support plays an important role in both abstinence and recovery from various addictions [61–63], some studies have shown mixed results regarding the role of social support in gambling [64]. A study by Savolainen et al. highlighted the role of offline support, showing that while offline support protects against problem gambling, online support is a risk for problem gambling [65]. On the other hand, some studies have shown mixed results regarding the role of offline peer support [66]. It may also be possible that high levels of social support indicate that a person is in distress and therefore seeks more support.

In the present study, adolescents were found to have psychosocial problems such as emotional, behavioural, peer, prosocial and hyperactivity problems. This suggests that there are important triggers of gambling addiction in adolescents. ‘Hyperactivity and attention deficit problems’ suggest that problem gamblers have difficulties with cognitive aspects (for exp. attention) that may be associated with lower school performance. This interpretation is consistent with prior evidence demonstrating that motor and cognitive impulsivity are among the strongest predictors of addiction tendency, even after controlling for emotion regulation variables [67].‘Problems coping with peers’ refers to the degree and quality of social adjustment within a social community. High scores for ‘behavioural problems’ indicate that problem gamblers are prone to impulsive behaviours and antisocial acts. This is consistent with findings that problem gambling is associated with an increased likelihood of committing crime [68, 69]. In accordance with the DSM-IV criteria, where antisocial acts are recognised as diagnostic criteria, it can be concluded that the development of problem gambling may promote subsequent offending.

Beyond depressive and anxiety symptoms, the broader psychosocial profile assessed by the SDQ warrants further consideration when interpreting gambling vulnerability among working adolescents. Although the primary analytical model emphasized depressive and anxiety symptoms as proximal correlates of gambling severity, the findings should also be interpreted within the broader psychosocial framework captured by the SDQ subscales. In particular, hyperactivity and conduct-related difficulties—reflecting impulsivity, poor inhibitory control, and behavioral dysregulation—are well-established risk factors for adolescent gambling involvement and may represent more direct pathways to gambling behavior than peer-related problems [56]. In contrast, peer problems are more likely to exert indirect or contextual influences by shaping social norms and exposure opportunities rather than directly driving gambling behavior [9]. This differentiation underscores the importance of addressing specific psychosocial dimensions, particularly impulsivity-related traits, when designing targeted prevention and intervention strategies for high-risk adolescents.

In the present study, according to our regression analysis results, we found that sociodemographic characteristics had a significant effect on adolescent gambling. Variables such as age, number of siblings, daily sleeping hours, number of cigarettes smoked, number of narghile smoked, internalised score (behavioural and hyperactivity problems) and externalised score (emotional and peer problems) of working children explained 87.3% of the change in gambling screening test scores (R2 = 0.873). It was found that internalised score (behavioural and hyperactivity problems) had the greatest effect on the gambling screening test score and age of the child had the lowest effect (Table 6).However, while age demonstrated a minimal linear effect (β = 0.05), post-hoc quadratic modeling suggested a significant non-linear relationship, with risk peaking during mid-adolescence (ages 16–17). Furthermore, this peak interacted significantly with weekly work hours, explaining an additional variance in gambling scores. In their study on the role of age, Sheela et al. found no significant relationship between age and gambling behaviour in adolescence [70], however, an earlier age at first gambling was identified as a risk factor [51]. McBride and Derevensky reported that a higher rate of non-gamblers were under 18 years of age [71] In a study by Rossen et al., it was reported that students with gambling habits reported significantly more mental health problems and other addictions/risk behaviours. Among high-risk behaviours, smoking has been reported to significantly increase the odds of developing gambling addiction [72]. In a study by Castrén et al., both smoking and drinking due to intoxication were significantly associated with being at risk and problem gambling compared to non-smokers and participants who did not drink due to intoxication [73]. Our results are consistent with the literature.

In line with recent bibliometric evidence highlighting thematic fragmentation and the lack of integrated frameworks in behavioral addiction research [74], the present findings contribute to the literature by contextualizing adolescent gambling within a psychological Consequences framework, particularly among socioeconomically disadvantaged working youth.

### Limitations

Although the present study was conducted with high methodological effort, it has some limitations. Due to the cross-sectional design, the results are limited to the measurement tools used. The results of the study can only be generalised to working adolescents. Furthermore, while key sociodemographic variables were modelled, the analysis of age as a linear predictor may have masked important non-linear developmental trends. Future longitudinal research is necessary to explore potential quadratic relationships, such as risk peaking in mid-adolescence, and to test for interactions with factors like work intensity, which could provide a more nuanced understanding of vulnerability across different developmental stages.

In addition to the cross-sectional design, the use of snowball sampling constitutes an important limitation. While this approach enabled access to a hard-to-reach population of working adolescents, it may have resulted in recruitment chains characterized by shared social networks and similar risk profiles, thereby increasing sample homogeneity. Such clustering may have amplified the observed prevalence of gambling and psychosocial difficulties and limits the representativeness of the findings. Furthermore, adolescents who were not connected to existing peer networks or who declined participation may have been underrepresented, potentially excluding subgroups with different gambling or psychosocial profiles. Consequently, the findings should not be extrapolated beyond socioeconomically disadvantaged, street-working adolescents in urban settings. Future studies employing probability-based or mixed sampling strategies are needed to strengthen generalizability and to disentangle network-related biases inherent in snowball recruitment.

The relatively high prevalence of pathological gambling observed in this study should be interpreted in light of the use of a non-probability (snowball) sampling method and the focus on a socioeconomically disadvantaged group of working adolescents, which limits the generalizability of the findings to the broader adolescent population.

## Conclusion

The study found that the vast majority of working adolescents surveyed exhibited pathological gambling behaviour, which is rooted in familial modelling (particularly by fathers) and compounded by work stressors, borrowing and theft. This behaviour explains 87.3% of the variance. This familial modelling, compounded by work stressors, is responsible for the 76.8% pathological gambling rate, highlighting the urgent need for integrated family-youth interventions. This gambling addiction is strongly influenced by internalised and externalised psychosocial problems, such as behavioural, emotional and hyperactivity issues, identified in the adolescents. It is also directly linked to factors such as depression, anxiety and perceived social support. On the other hand with psychosocial factors explaining 87% variance, advocate routine SDQ integration in labor registries for early diversion, potentially averting generational cycles.

Based on the study's future-oriented recommendations, it is suggested that intervention programmes be designed using a tiered approach. Accordingly, universal, school-based programmes should support prosocial skills, and targeted cognitive behavioural therapy (CBT) should be provided to high-risk youth identified via the Strengths and Difficulties Questionnaire (SDQ). Family therapy interventions that address parental modelling should also be developed. This multi-tiered, comprehensive approach will effectively reduce the impact of depression and anxiety on gambling addiction in adolescents by strengthening social support mechanisms and enhancing coping strategies.

In addition to individual and family-level interventions, the findings highlight the necessity of broader social and policy-level responses. The high prevalence of gambling addiction among working adolescents should be interpreted within the intersecting contexts of child labor, poverty, and limited access to education and social protection. Therefore, public health strategies should incorporate social policies aimed at reducing child labor, supporting economically disadvantaged families, and strengthening child protection and welfare systems. Addressing these structural determinants is essential to mitigate long-term public health risks and to prevent the intergenerational transmission of gambling-related vulnerability.

## Data Availability

Data or information needed to re-produce the findings presented are available from the corresponding author upon reasonable request.

## Abbreviations

OR: Odds ratio
AMOS: Analysis of Moment Structures
CMIN: Chi-Square Minimum
RMSEA: Root Mean Square Error of Approximation
CFI: Comparative Fit Index
NFI: Normed Fit Index
GFI: Goodness of Fit Index
IFI: Incremental Fit Index
RFI: Relative Fit Index
TLI: Tucker-Lewis Index
VIF: Variance Inflation Factor
